# Design and Fabrication of a Real-Time Measurement System for the Capsaicinoid Content of Korean Red Pepper (*Capsicum annuum* L.) Powder by Visible and Near-Infrared Spectroscopy

**DOI:** 10.3390/s151127420

**Published:** 2015-10-29

**Authors:** Jongguk Lim, Giyoung Kim, Changyeun Mo, Moon S. Kim

**Affiliations:** 1 National Institute of Agricultural Science, Rural Development Administration, 310 Nongsaengmyeng-ro, Wansan-gu, Jeonju, Jeo1labuk-do 54875, Korea; E-Mails: limjg@korea.kr (J.L.); cymoh100@korea.kr (C.M.); 2 Environmental Microbiology and Food Safety Laboratory, BARC-East, Agricultural Research Service, US Department of Agriculture, 10300 Baltimore Avenue, Beltsville, MD 20705, USA; E-Mail: Moon.Kim@ars.usda.gov

**Keywords:** visible and near-infrared, spectroscopy, capsaicinoid content, red pepper powder, partial least square regression

## Abstract

This research aims to design and fabricate a system to measure the capsaicinoid content of red pepper powder in a non-destructive and rapid method using visible and near infrared spectroscopy (VNIR). The developed system scans a well-leveled powder surface continuously to minimize the influence of the placenta distribution, thus acquiring stable and representative reflectance spectra. The system incorporates flat belts driven by a sample input hopper and stepping motor, a powder surface leveler, charge-coupled device (CCD) image sensor-embedded VNIR spectrometer, fiber optic probe, and tungsten halogen lamp, and an automated reference measuring unit with a reference panel to measure the standard spectrum. The operation program includes device interface, standard reflectivity measurement, and a graphical user interface to measure the capsaicinoid content. A partial least square regression (PLSR) model was developed to predict the capsaicinoid content; 44 red pepper powder samples whose measured capsaicinoid content ranged 13.45–159.48 mg/100 g by per high-performance liquid chromatography (HPLC) and 1242 VNIR absorbance spectra acquired by the pungency measurement system were used. The determination coefficient of validation (*R_V_^2^*) and standard error of prediction (*SEP*) for the model with the first-order derivative pretreatment method for Korean red pepper powder were 0.8484 and ±13.6388 mg/100 g, respectively.

## 1. Introduction

Red peppers (*Capsicum annuum* L.) are an annual plant that belongs to the *Solanaceae* family, native to South America. They are widely grown in tropical to temperate climates and have been important crop in Korea for a long time. Red pepper powders made by drying and grinding red-ripened red peppers are the main ingredient in Korean traditional foods such as Kimchi (fermented cabbage) and Gochujang (spicy red pepper paste) [1,2]. The pungent components in red pepper are collectively called capsaicinoids, and such pungent components are regarded to cause a feeling of pain when the taste nerve is strongly stimulated, rather than a sense of taste; these components can cause severe pain when they come in contact with skin [3]. Among the capsaicinoid components of red pepper account, capsaicin (C_18_H_27_NO_3_) and dihydrocapsaicin (C_18_H_29_NO_3_) account for 80% to 90% of the total pungency, and other components such as nordihydrocapsaicin (C_17_H_27_NO_3_), homocapsaicin (C_19_H_29_NO_3_), and homodihydrocapsaicin (C_19_H_31_NO_3_) are homologs [4,5,6,7]. Uniformity and scale of hotness are main criteria for grading quality of red pepper powder. To produce high quality red pepper powder and increase farmer’s income, measurement of the capsaicinoids content of red pepper is necessary.

One of the conventional methods to measure the pungency level or capsaicinoid concentration of red pepper powder is the Scoville Organoleptic Test [8]. This method was developed by American pharmacist Wilber Scoville. In this method, red pepper solution is diluted with sugar water until the pungency cannot be detected by five taste panels, and the final dilution level becomes the Scoville Heat Unit (SHU) value. However, this method is prone to producing varied results depending on evaluating panel's subjectivity and thus does not ensure objective results; hence, this method is used only as brief method to measure pungency [9,10]. The most trusted and accurate method to determine the capsaicinoid content and pungency of red pepper is through chemical assays. Examples of such assays are the high performance liquid chromatography (HPLC), gas chromatography (GC), high performance liquid chromatography-mass spectrometry (HPLC-MS), and gas chromatography–mass spectrometry (GC–MS) [11,12,13,14]. However, these methods have the disadvantages that they require tedious sample preprocessing, take a long time for analysis, and only professional operators can perform analyses by this method [2]. 

Several researchers have explored alternative methods to measure the capsaicinoid concentration of red pepper powder easily and conveniently such as techniques using carbon nanotubes (CNTs), electronic nose, and near-infrared spectroscopy (NIRS) [11,15,16]. Among these methods, NIRS showed feasibility as a technique to measure pungency nondestructively without additional chemical preprocessing of samples [17,18,19,20]. 

**Figure 1:**
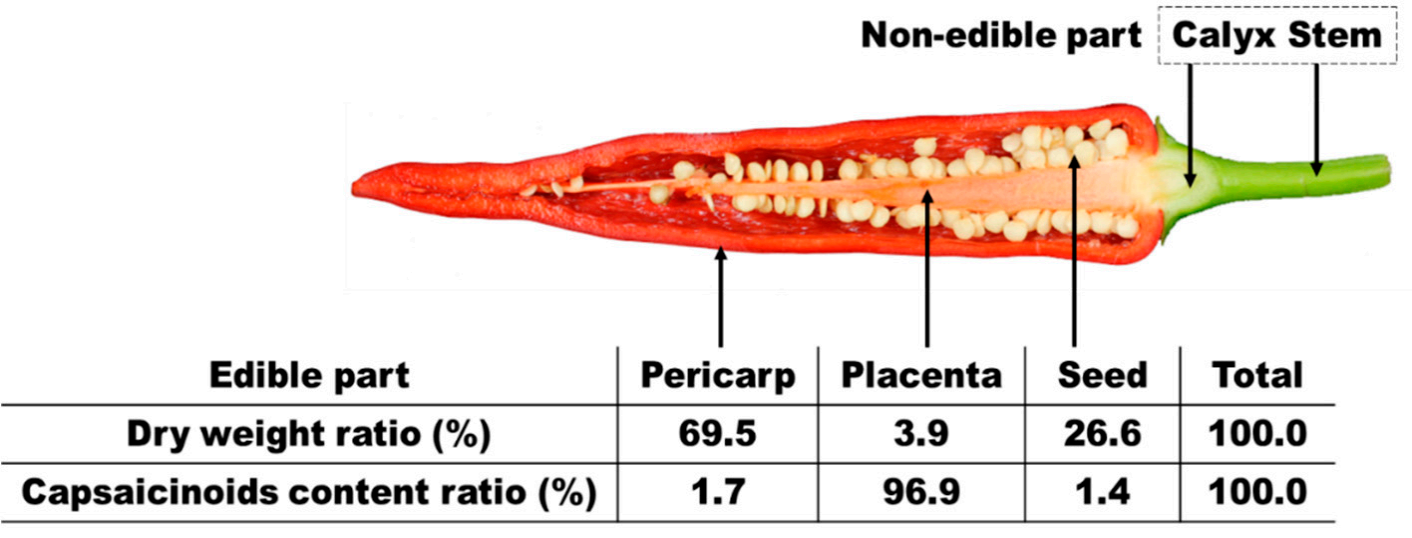
Ratio of the weight and capsaicinoid content for each part of dried Korean red-pepper (*Capsicum annuum* L.).

Rice flour, wheat flour, or soy flour produced by grinding cereals consist of relatively uniform materials, whereas red pepper powder made by grinding red pepper contain the pericarp, seed, and placenta, all of which have different properties [21]. The capsaicinoids in red pepper are produced and concentrated mostly in the placenta, though in some varieties, some of the capsaicinoid components are also found in the pericarp and seed [22,23,24]. Figure 1 shows the pungency distribution of red pepper along with the relation between the dry weight and capsaicinoid content ratio in each part of dried red pepper; the information indicates that the placenta that accounts for only 3.9% of the total weight contains almost 96.9% of all capsaicinoids, which form the pungency component. This means that chemical assays such as HPLC and GC analysis using a small sample amount or the commercial NIRS that takes measurements at only one point may show large deviations in pungency depending on the location, distribution, and inclusion of placenta in red pepper powders. Thus, it is necessary to improve pungency measurement accuracy by scanning a large number of points continuously to minimize the deviation in pungency caused by the influence of the placenta in red pepper powder. Furthermore, uneven compact levels or surfaces in red pepper powders may correspond to different scatter levels and reflectance spectra even in the same sample, and hence, equipment that can form surfaces uniformly consistently and automatically is required. 

Thus, in this research, a surface-scanning-type pungency measurement system that can measure the pungency of red pepper powder continuously by visible and near infrared (VNIR) spectroscopy in the effective wavelength range of 450 nm to 950 nm was designed and fabricated. We developed the critical equipment that can scan red pepper powder continuously to reduce the deviation in pungency caused by the influence of the placenta and also developed devices that can make the red pepper powder surface uniform in order to enable acquiring stable reflectance spectra. In addition, the capsaicinoid content prediction performance of the developed pungency measurement system was validated by using Korean red pepper powder samples.

## 2. Design and Fabrication 

### 2.1. System Design 

**Figure 2:**
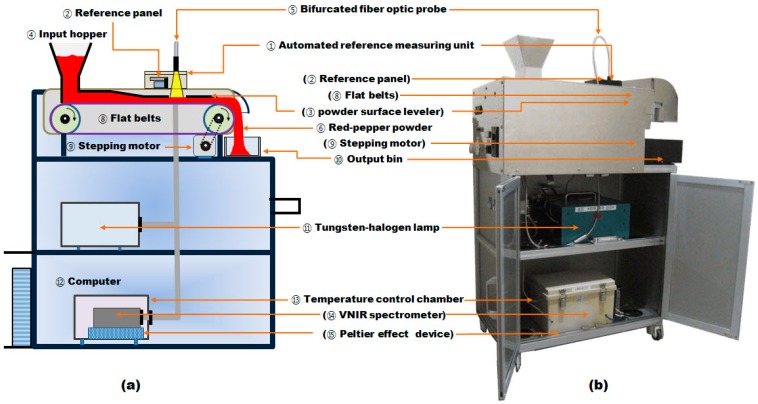
A schematic diagram (**a**) and photo (**b**) of the measurement system for the capsaicinoid content of red pepper powder.

The developed system aimed to scan the well-leveled powder surface continuously to minimize influence of the distribution of the placenta, thus acquiring stable and representative spectra. To achieve these functions, the system was designed to have a sample input hopper through which the red pepper powder was introduced into the system; a stepping motor and flat belts to convey the inputted red pepper powder; a powder surface leveler to realize uniform shape of the conveyed red pepper powder surface; a standard reference panel and an automated reference measuring unit to acquire the standard reflectance spectrum and check whether the illumination is correct; a bifurcated fiber optic probe to supply light from a 100-W tungsten-halogen lamp and to receive light reflected from the sample using the VNIR spectrometer; a temperature control chamber in which the Peltier effect device was mounted to maintain a constant working temperature for the VNIR spectrometer;, and a computer to perform system control and to operate programs to predict the capsaicinoid content. Figure 2a shows an overall schematic diagram of the measurement system for the capsaicinoid content of red pepper powder.

### 2.2. Critical Parts

#### 2.2.1. Visible and NIR Spectrometer

**Figure 3:**
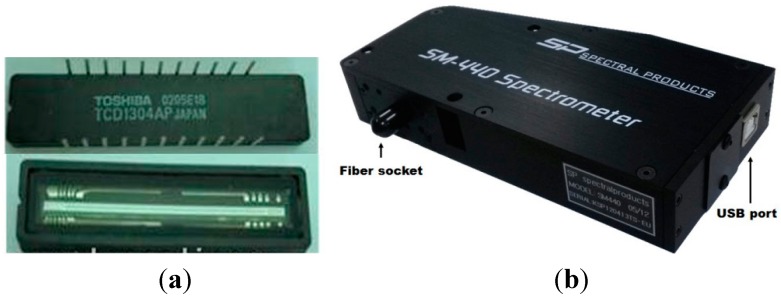
A photograph of the charged-couple device (CCD) linear image sensor (**a**) and spectrometer (**b**) used for measuring the spectrum of red-pepper powder.

The VNIR spectrometer mounted to the measurement system contains a charged-couple device (CCD) linear image sensor (TCD1304AP, Toshiba Corporation, Tokyo, Japan), as shown in Figure 3a. The CCD image sensors consist of 3648 pixels of dimensions 8 × 200 μm per sensor, with a pixel interval of approximately 0.25 nm. Figure 3b shows the spectrometer (SM440, Korea Spectral Products, Seoul, Korea) with the embedded CCD image sensors for a wavelength range of 145–1176 nm and a slit size of 50 μm. The data interface communicates via a Universal Serial Bus (USB), and the optic fiber probe coupler is coupled with a subminiature A (SMA-905) connector or fiber coupler (FC) standard. The main specifications are listed in Table 1. 

**Table 1:** Specifications of the CCD image sensor.

Detector	Number of pixels: 3648 Sensing pixel size: 8 × 200 μm
Computer interface	USB 1.1/2.0 16 bit 500 kHz
Slit	50 μm
Fiber coupler	SMA 905/FC standard
Spectral rage	145–1176 nm
Dimensions	142 × 70 × 22 nm
Weight	408 g

**Figure 4:**
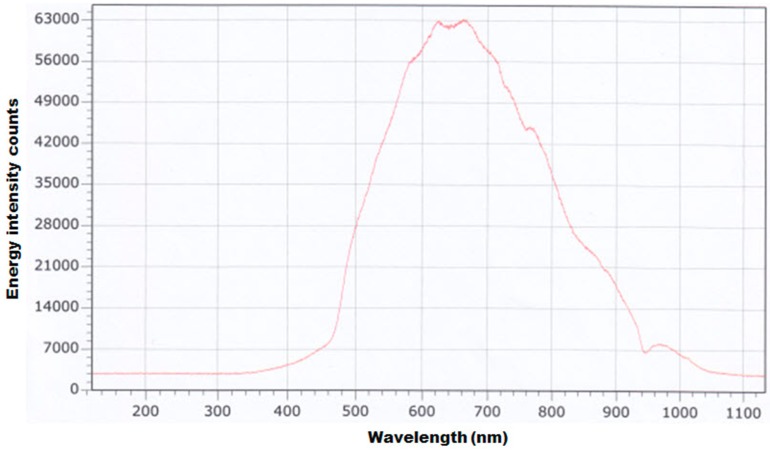
The charged-couple device (CCD) image sensor response for the 5-W tungsten-halogen light source.

Figure 4 shows the CCD spectrometer response measured by setting the integration time to 3500 ms by directly connecting the optic fiber probe to a 5-W tungsten-halogen lamp (ASB-W-005 5W, Korea Spectral Products) in terms of relative energy intensity (counts). The CCD spectrometer showed upper and lower direction peak characteristics at 620(↑), 630(↓), 670(↑), 760(↓), 770(↑), 950(↓), and 970(↑) nm wavelengths, and the maximum energy peak showed 63,800 counts at 667.6 nm. Thus, this research found that there is the need to consider such the characteristics of the spectrometer and light source. The VNIR spectrometer was embedded in a temperature control chamber using the Peltier effect device that is cooled or heated by a thermoelectric to eliminate unsuitable spectra and noise due to changes in the external temperature. The VNIR spectrometer to which the temperature control chamber was attached was mounted inside a closed box to maintain the working temperature at 25 °C.

#### 2.2.2. Light Source and Fiber Optic Probe

**Figure 5:**
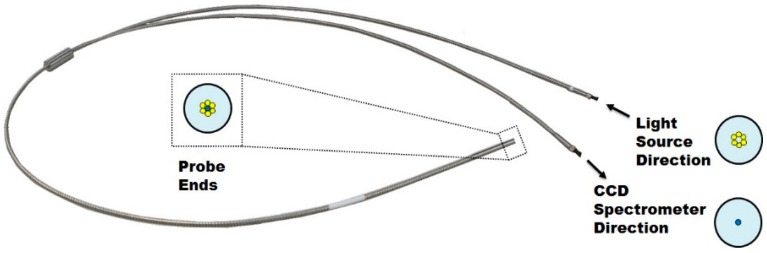
A photo of the bifurcated fiber optic probe used for reflectance measurement with the visible and near infrared spectrometer.

The light source used to illuminate the red pepper powder was a 100-W tungsten-halogen lamp (ASBN-W-100F-L, Korea Spectral Products, Seoul, Korea). The used wavelength range was 300–2600 nm, and the outputted illumination was 2000 lumens; the guaranteed lamp life time was 2000 h. Figure 5 shows the optic fiber probe (RP060-SMFR-U20-NS-BX, Korea Spectral Products, Seoul, Korea) that transmits the light outputted from the lamp and receives the light reflected from the red pepper powder. The probe used was a bifurcated fiber optic probe; the diameter of one optic fiber was 600 μm, and it was shaped in the form of a Y and was 2 m long.

At the ends of the probe, six optic fibers are arrayed outward to illuminate the red pepper powder, and at the center of the probe, there is an optic fiber that receives the light reflected from the red pepper powder and transmits it to the CCD spectrometer. Here, the wavelength range that can be transmitted by the optic fiber probe is 200–1100 nm.

#### 2.2.3. Powder Surface Leveler 

**Figure 6:**
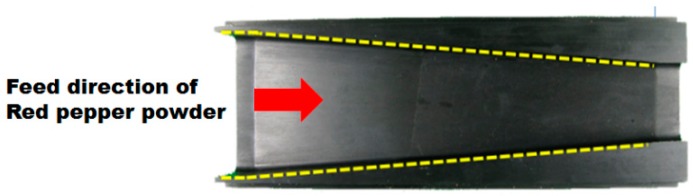
A photo of the powder surface leveler used to shape the surface evenly.

Powdered samples such as red pepper powder should have a constant compact level and even surface for the reflectance spectra to be stable. In this research, the powder surface leveler was mounted on the upper side in the middle of the flat belts in the direction of motion of the red pepper powder. The leveler made uniform the surface of the red pepper powder surface that was input into the sample input hopper. The powder surface leveler, when viewed from one side, had a slope as shown in Figure 2a, while it was trapezoidal, as seen in the top shown in Figure 6; the width of the trapezoid became smaller in the direction of motion of the red pepper powder. Thus, the red pepper powder located on the flat belts through the input hopper was passed through a narrow pipeline of the unit that makes the surface uniform as it moved, and the powder density was maintained while making the surface even. The conveying speed of the red pepper powder passing through the powder surface leveler was set at 14.3 mm/s by adjusting the speed of the flat belt by using the stepping motor.

#### 2.2.4. Automated Reference Measuring Unit

**Figure 7:**
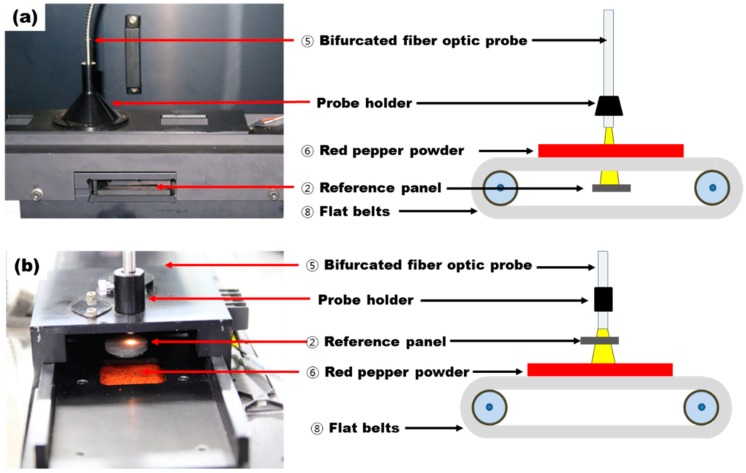
Photos comparing the standard reference in the automated reference-measuring unit to prevent the red pepper powder from contaminating mounting the reference in the lower part in the initial device (**a**) and mounting the reference in the upper part in the improved device (**b**).

The automated reference measuring unit employed a gray-type standard reference panel (SRS-05-020, Labsphere Inc., North Sutton, NH, USA) whose reflectivity factor was 0.781 to inspect whether the CCD spectrometer worked correctly and to check the acquisition status of a stable reflectance spectrum by setting its diameter as 20 mm. In addition, the standard reference was used to convert the acquired reflectance spectra into absorbance spectra. The reference panel performed reference measurements forcefully by means of the solenoid valve prior to the acquisition of the reflectance spectra of the red pepper powder. The automated reference-measuring unit was initially fabricated such that it was mounted on the lower part of the flat belts that convey red pepper powder, as shown in Figure 7a. However, contamination of the reference occurred because of scattered or fallen red pepper powders. To solve this problem, as shown in Figure 7b, the reference measurement location was changed to the upper part of the flat belts to prevent contamination of the reference panel by dropping red pepper powder, thus ensuring a reliable standard spectrum. 

#### 2.2.5. Interface Program

**Figure 8:**
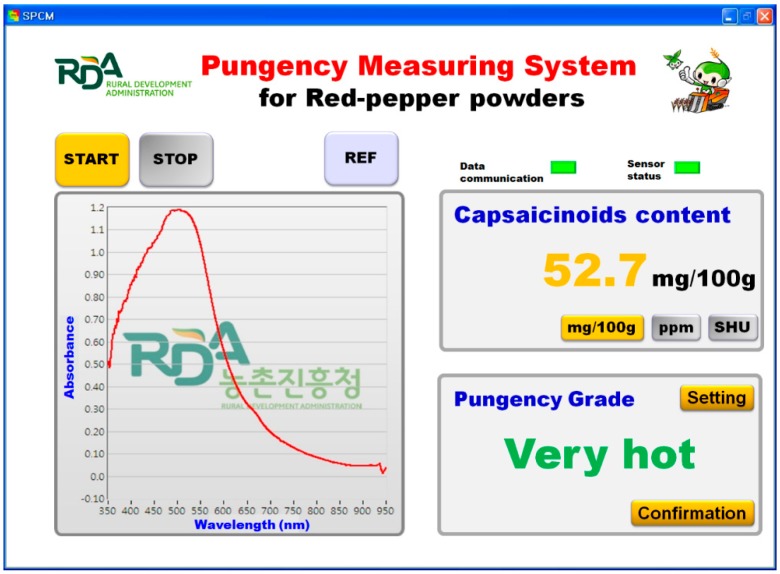
A screen shot of the graphical user interface for the measurement system for the capsaicinoid content of red pepper powder.

The interface program for system operation, reflectance spectra acquisition, and capsaicinoid content prediction was performed by a Windows-based in-house software developed using LabVIEW (v8.5, National Instruments Corp., Austin, TX, USA) with the software development kit (SDK) provided by the VNIR spectrometer manufacturer. Figure 8 shows the graphical user interface (GUI); its main functions are to calibrate the standard reflectivity of the standard reference and to display the capsaicinoid content in real time by loading the model developed to predict the pungency of red pepper powder by using the automated reference measuring unit. The capsaicinoids content display can be customized by users to show the values in mg% (mg/100 g), SHU, or ppm, and a pungency grade can also be set up by inputting a range in advance by users. The reflectance spectra acquired from the red pepper powder were pretreated using the Savitzky–Golay smoothing method with a gap of 6.5 nm to minimize the noise generated in the spectrometer. 

### 2.3. Integration of Components 

Figure 2b shows the real-time measurement system for the capsaicinoid content of red pepper powder. The overall operation process is as follows: once power is supplied to the system, the computer, tungsten halogen lamp, VNIR spectrometer, temperature control chamber, and Peltier effect device; subsequently, the pungency measurement program is run, as shown in Figure 8. The light transmitted from the light source illuminates the red pepper powder surface through the bifurcated fiber optic probe, and the reflected light is delivered to the VNIR spectrometer. The reference panel in the automated reference-measuring unit checks for any abnormality in VNIR spectrometer and acquires the standard reflectance spectrum. The stepping motor drives the flat belts and inputs the red pepper powder sample into the input hopper. The inputted red pepper powder is passed through the light illumination unit as its surface becomes even, and the reflectance spectra measured is converted into absorbance spectra for measuring the capsaicinoid content of the red pepper powder. The result is displayed on the monitor, as shown in Figure 8, and the red pepper powder is discharged to the output bin. 

### 2.4. Experimental Setup

**Figure 9:**
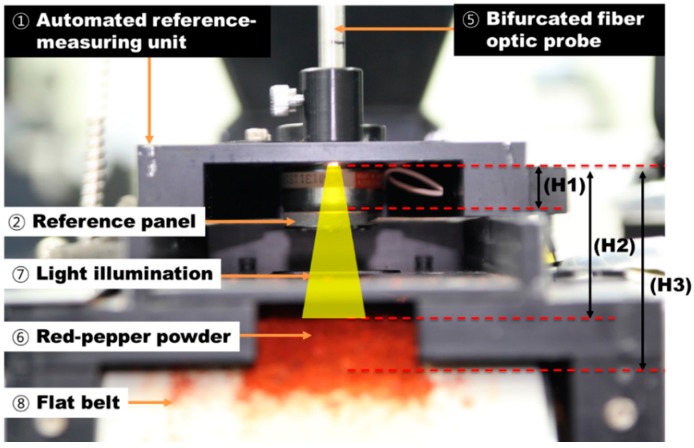
H1: End of optic probe to reference panel surface; H2: End of optic probe to red pepper powder surface; H3: End of optic probe to conveyor belt surface.

**Figure 10:**
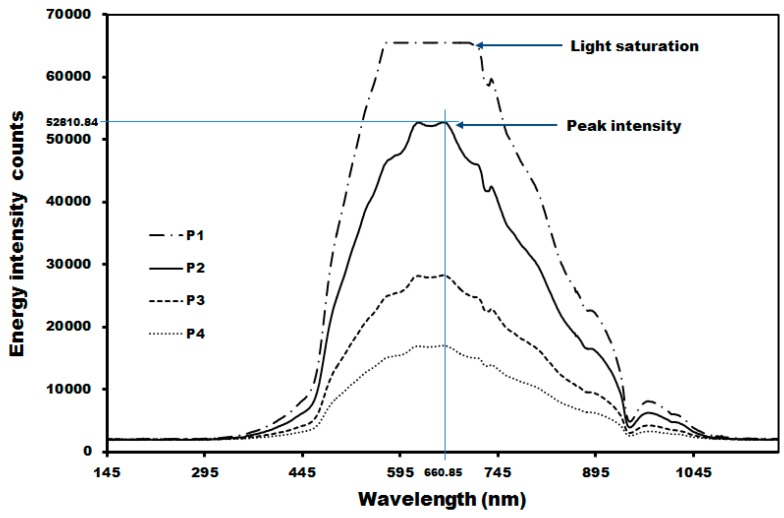
Changes in energy intensity in the red pepper powder spectrum due to differences in the probe positions.

Figure 9 shows the interior of the automated reference-measuring unit in the real-time pungency measurement system for red pepper powder. The reference panel is operated by means of the closed solenoid valve normally. The light transmitted through the bifurcated fiber optic probe has a divergence angle of 12.7° at the end of the probe. Here, the light arrival distance and field of view (FOV) up to the reference panel, the red pepper powder surface, and the flat belts from the end of the optic fiber probe can vary. In Figure 10, the distance from the probe end to the surface of the reference panel is indicated by H1, that to the red pepper powder surface is indicated by H2, and that to the bottom surface of the flat belts is indicated by H3. 

Figure 10 shows the energy intensity counts per distance to determine the probe height to realize the maximum reflectivity intensity and reliable spectrum acquisition at H1 position where the reference panel is installed. P1, P2, P3, and P4 are spectra depends on the distance to end of optic probe to red pepper powder surface. Here, H1, H2, and H3, gap distances, and peak intensity values at P1, P2, P3, and P4 are listed in Table 2. At P1, light saturation occurred, while P3 and P4 had relatively weak energy. Thus, P2 position was selected considering the light saturation and energy intensity. 

As shown in Table 2, as the distance from the probe end where light was incident increased, the FOV area increased but the reflectivity intensity weakened. That is, as the optic fiber probe approached the red pepper powder, the light intensity and reflectance spectrum intensity increased, whereas the FOV where the light was incident on the surface of red pepper powder decreased. 

**Table 2:** Different peak intensities and field of view (FOV) area depending on probe position of quality factor measurement system for red pepper powder.

Spectrum Types	Distance to Probe End	FOV (mm^2^)	Distances (mm)	Peak Intensity
P1	H1	4.9	2	65,538
	H2	56.7	25	
	H3	122.7	34	
P2	H1	18.1	6	52,810
	H2	102.1	29	
	H3	153.9	38	
P3	H1	22.1	9	28,330
	H2	128.7	32	
	H3	193.6	41	
P4	H1	28.3	12	17,050
	H2	143.1	35	
	H3	219.0	42	

**Figure 11:**
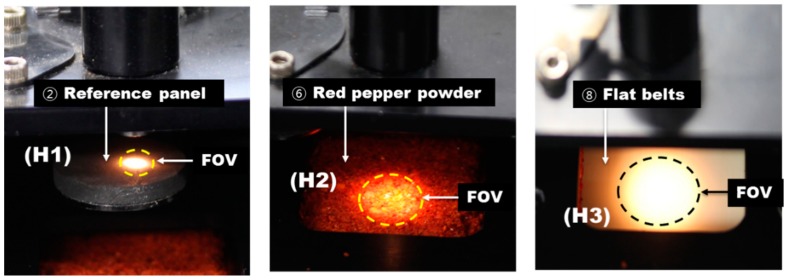
Shape of the incident light spot on the surface of the reference panel (H1), red pepper powder (H2), and flat belts (H3).

**Figure 12:**
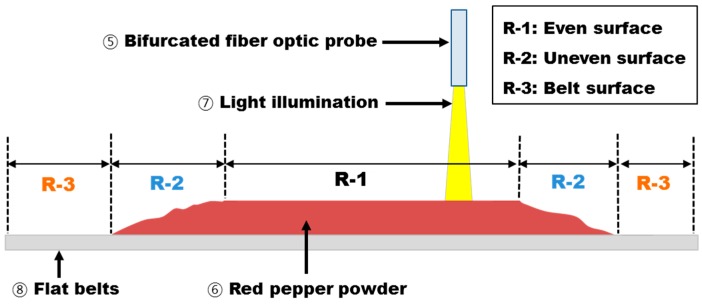
A schematic view of the condition for absorbance spectra acquisition.

Figure 11 shows the light incident on the reference panel surface, red pepper powder surface, and up to the flat belts surface from P2, which was set as the optimum position. Here, the distance from the optic fiber probe end to the reference panel was 6 mm, and the FOV area was 18.1 mm^2^. The wavelength at which 52,810.84 (counts), *i.e.*, the maximum reflectivity intensity, was measured was 660.85 nm. The target region for measurement of absorbance spectra was divided into three regions depending on the shape of the samples as shown in Figure 12: the first region (R-1) was where the surface-leveled red pepper powder surface was passed; the second region (R-2) was where the red pepper powder surface was shaped unstably before and after the red pepper powder surface was shaped evenly; and the third region (R-3) was where the white-color absorbance spectra of the flat belts were acquired without red pepper powder. 

**Figure 13:**
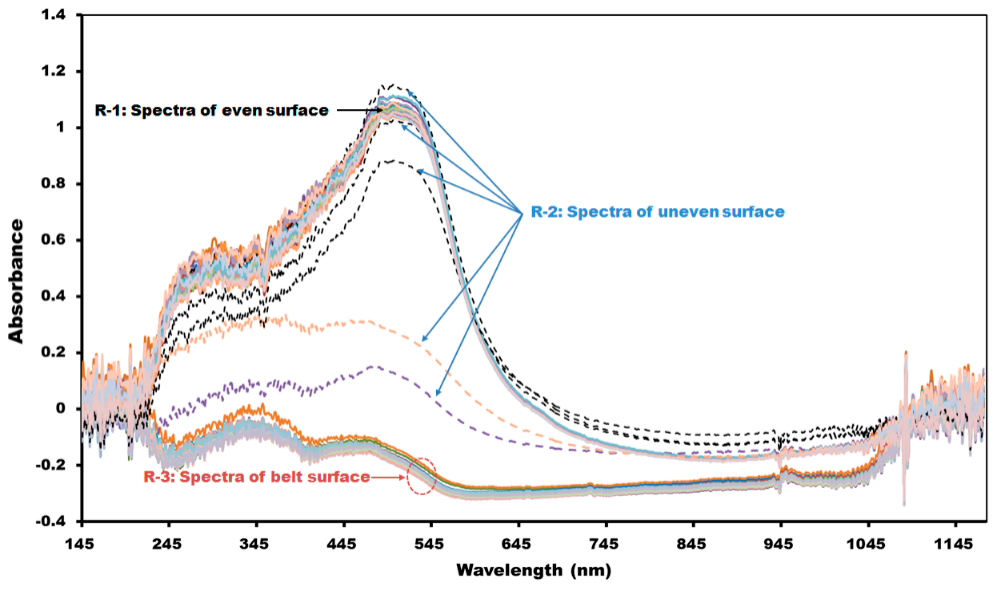
Spectrum shapes measured from the even or uneven surface of red pepper powder and from the belt surface.

Figure 13 shows the absorbance spectrum of R-1, R-2, and R-3 regions; the absorbance spectrum of even red pepper powder surface (R-1) had an absorbance intensity as high as 1, and the absorbance spectrum of the flat belts (R-3) had an intensity of 0 or less. That is, red pepper powder had higher light absorbance compared to that of the flat belts, and the unnecessary flat belt spectra could be removed during the pungency prediction algorithm development by using the absorbance intensity difference in a specific wavelength range. The threshold value applied here was set such that the absorbance spectra below 0.5 of absorbance ratio in the 500 nm band was removed from the effective spectrum. 

However, as shown in Figure 13, the spectra in the R-2 region where the red pepper powder surface was shaped unevenly could not be removed because it was measured similarly as in the case of the even surface spectra; hence, an additional method was implemented to filter the abnormal spectra. First, as shown in Figure 13, the spectra that were suspected to be the absorbance spectra measured at the uneven red pepper powder surface in R-2 region in the first half and second half among the total spectra acquired sequentially from these regions were removed. Since 0 to 2 times of the spectra were measured in R-2 region, two spectra of the first half and second half were removed from the total spectra acquired for developing the algorithm. Another method is as follows: out of the spectra acquired in R-1 region, if that for red pepper powder surface is removed, they show different absorbance intensity from normal spectra; they are compared with the average values of the total normal spectra in the program to select the spectra that has a higher or smaller value than the set standard value to remove them.

## 3. Experimental Results

A test was conducted to measure the pungency of Korean red pepper powders and to validate the prediction performance using the developed system. Forty-four red pepper powder samples produced in Chungcheongbuk-do Goesan area in Korea from 2009 to 2013 were used. The standard ASTA analysis method for measurement of capsaicinoids content was used [25]. To quantify the real values of the capsaicinoid contents, which are the pungency components of red pepper powder, the HPLC (YL9000, Younglin, Anyang, Korea) analysis method was used. Contents of capsaicin (M-2028, Sigma, St. Louis, MO, USA) and dihydrocapsaicin (M-1022, Sigma, St. Louis, MO, USA), which accounted for 90% or more of the pungency components in red pepper powder, were summed using the standard materials to produce calibration curves with regard to the capsaicinoid contents. The total capsaicinoid content was analyzed to be in a range of a minimum of 13.45 mg/100 g to a maximum of 159.48 mg/100 g. Of the red pepper powders produced in Goesan area, 82% samples (36/44) had less than 60 mg/100 g of capsaicinoid contents.

The effective wavelength range used to predict red pepper powder capsaicinoid content was set between 450 nm and 950 nm. A total of 1242 reflectance spectra acquired with the 44 red pepper powder samples using three iterations of tests, and the spectra were converted to absorbance spectra ($A_{\lambda }$) using Equation (1) [26]:

(1) $$A_{\lambda }=\log_{10}\left(\frac{R_{\lambda }-D_{\lambda }}{S_{\lambda }-D_{\lambda }}\right)$$

Here, *λ* is the given wavelength; *S_λ_* is the raw reflectance intensity at *λ*; *D_λ_* is the reflectance intensity of the dark reference at *λ*; and *R_λ_* is the reflectance intensity of the standard reference at *λ*. Using the 1242 converted absorbance spectra, the partial least square regression (PLSR) prediction model was developed. The performance of the PLSR prediction models could be evaluated by how well the predicted values in the developed model could predict the numerically measured values. Such performance evaluation factors are expressed by various methods, and the determination coefficient of calibration (*R_C_^2^*) and the standard error of calibration (*SEC*) in the developed prediction models represent the linear fitness between the predicted and the measured values. *R^2^* and *SEC* are defined as shown in Equations (2) and (3):

(2) $$R^{2}=\;\frac{\sum_{i=1}^{n}{\left(y_{c_{i}}-\bar{y}\right)}^{2}}{\sum_{i=1}^{n}{\left(y_{i}-\bar{y}\right)}^{2}}$$

(3) $$SEC=\sqrt{\frac{\sum_{i=1}^{n}{\left(y_{i}-y_{c_{i}}\right)}^{2}}{n-p-1}}$$

Here, $y_{i}$ are the measured values; $y_{c_{i}}$ is the value predicted by the calibration equation; $\bar{y}$ is the mean value of $y_{i}$; *n* is the number of samples in the calibration set; and *p* is the number of independent variables in the regression optimized by cross validation. Full cross validation was performed for the developed models to validate the prediction performance. Cross validation is a method of validating how accurately a model can represent the measured samples by removing the existing measured samples one by one to construct a model and by finding the difference with the model's response value with respect to the total number of samples. The developed validation model can be evaluated with the standard error of prediction (*SEP*) of the calculated prediction model by reflecting the bias, which is the difference between the measured and predicted values in addition to the determination coefficient of validation (*R_V_^2^*), which are expressed as shown in Equations (4) and (5):

(4) $$bias=\frac{\sum_{i=1}^{n}{\left(y_{i}-\bar{y_{v_{i}}}\right)}^{2}}{n}$$

(5) $$SEP=\sqrt{\frac{\sum_{i=1}^{n}{\left[\left(y_{i}-y_{v_{i}}\right)-bias\right]}^{2}}{n-1}}$$

Here, $y_{i}$ are the measured values; $y_{v_{i}}$ are the values predicted by the validation equation; and *n* is the number of samples in the validation set. 

**Figure 14:**
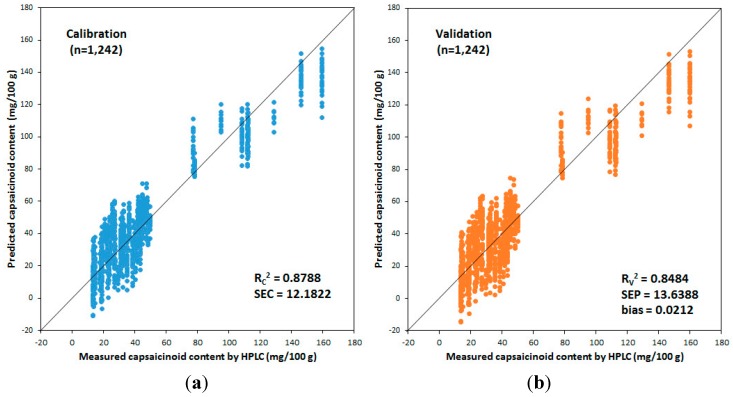
Calibration (**a**) and validation (**b**) results of the PLSR model in which the first-order derivative pretreatment method was applied to predict the pungency of red pepper powder produced in Geosan area in Korea using the real-time measurement system for the capsaicinoid content of red pepper powder.

Figure 14 shows the results of the developed calibration PLSR model and validation PLSR model with a PLS factor 9 by applying the first-order derivative pretreatment method; the best prediction performance among the developed PLSR models was thus determined. In the graph, the horizontal axis shows the real capsaicinoid content values using the HPLC, and the vertical axis shows the capsaicinoid content of the predicted red pepper powder using the developed real time measurement system in this research. The calibration performance showed that *R_C_*^2^ = 0.88 and *SEC* = ±12.18 mg/100 g, while the validation performance showed that *R_V_*^2^ = 0.85, *SEP* = ±13.64 mg/100 g, and bias = 0.02, which indicated good performance. The performance of the PLSR model for the prediction of capsaicinoid content in the red pepper powder was improved than the results developed in the past [20].

## 4. Conclusions and Outlook

In this research, a system was designed and fabricated for measuring the capsaicinoid content of red pepper powder; the system can determine the pungency of red pepper powder in a non-destructive and rapid manner by using a VNIR spectrometer with an effective wavelength range 450 nm to 950 nm. To overcome the disadvantages and limitations resulting from the use of existing chemical assay methods and commercial NIRS, we developed equipment that can acquire the reflectance spectra of red pepper powder samples continuously. The main equipment of the pungency measurement system consisted of a VNIR spectrometer that acquired the reflectance spectra, a 100-W tungsten-halogen lamp, and a bifurcated fiber optic probe. The system had a powder surface leveler to make the surface of the red pepper powder even and uniform, and also an automated reference measuring unit to apply the standard reference for conversion to absorbance spectra and for checking for abnormalities in the light source and VNIR spectrometer. An interface program was developed to perform system operation, spectra acquisition, and capsaicinoid content prediction, and by integrating all the units, the measurement system for capsaicinoid content of red pepper powder was fabricated. The optimal conditions of each unit were conducted tests to identify, and an algorithm was developed to acquire the absorbance spectra of pure red pepper powder. Using the developed system, a PLSR model for capsaicinoid content prediction was developed using 1242 absorbance spectra acquired from 44 samples of red pepper powder produced in Chungcheongbuk-do Goesan area in Korea. The best prediction performance with a PLS factor of 9 was shown by the PLSR model developed using the first-order derivative pretreatment method, and the validation of the PLSR model showed that *R_V_^2^* = 0.85, *SEP* = ±13.64 mg/100 g, and *bias* = 0.02, indicating showed good performance. Tests for performance validation showed that though the capsaicinoid contents of the samples range were 60 mg/100 g or lower, samples with wider pungency range will be obtained to develop the PLSR model in the future to improve the prediction performance. The developed system has temporal resolution high enough to measure the capsaicinoid contents of red pepper powder in real time; its feasibility for use in commercial systems in the red pepper powder processing center where pungency grading is done was proved. Early utilization of the developed system in agriculture can be facilitated by realizing further reduction in the fault rate of pungency grading through periodical calibration and improvement in the prediction model by using large volumes of accumulated spectral data. The platform presented in this paper is novel and can be used for analyzing other powder samples in agricultural and pharmaceutical fields.
